# The Evolutionary Interplay of Somatic and Germline Mutation Rates

**DOI:** 10.1146/annurev-biodatasci-102523-104225

**Published:** 2024-07-24

**Authors:** Annabel C. Beichman, Luke Zhu, Kelley Harris

**Affiliations:** 1 Department of Genome Sciences, University of Washington, Seattle, Washington, USA; 2 Department of Bioengineering, University of Washington, Seattle, Washington, USA; 3 Computational Biology Division, Fred Hutchinson Cancer Center, Seattle, Washington, USA

**Keywords:** germline mutation, somatic mutation, drift-barrier hypothesis, mutator allele, Peto’s paradox, aging, effective population size

## Abstract

Novel sequencing technologies are making it increasingly possible to measure the mutation rates of somatic cell lineages. Accurate germline mutation rate measurement technologies have also been available for a decade, making it possible to assess how this fundamental evolutionary parameter varies across the tree of life. Here, we review some classical theories about germline and somatic mutation rate evolution that were formulated using principles of population genetics and the biology of aging and cancer. We find that somatic mutation rate measurements, while still limited in phylogenetic diversity, seem consistent with the theory that selection to preserve the soma is proportional to life span. However, germline and somatic theories make conflicting predictions regarding which species should have the most accurate DNA repair. Resolving this conflict will require carefully measuring how mutation rates scale with time and cell division and achieving a better understanding of mutation rate pleiotropy among cell types.

## INTRODUCTION

One of life’s more astounding features is the fact that a single genome can encode so many cell types with disparate structures, functions, and metabolic processes. Despite this intraorganismal diversity, there are a few basic housekeeping tasks that all cells have in common, with perhaps the most fundamental being the maintenance and dissemination of the genome itself. Even post-mitotic cells must avoid harmful mutation loads that might interfere with the production of the proteins the cells will need for the duration of their lives (1), and actively replicating cells are held to the arguably higher standard of passing on copies of the genome that are capable of encoding whole organisms over the course of many generations to come (2–5). But how many mutations is each cell and each organism able to tolerate, depending on its function and life history, and how do genetics and gene regulation keep mutation rates within these limits? This question has long captured the attention of evolutionary biologists (6–10), but it is only within the past decade that rigorous measurements of mutation rates have begun to provide a foundation of relevant data in multicellular organisms (11–13).

Every genome provides for its own maintenance and dissemination by encoding DNA repair proteins, as well as high-fidelity polymerases that proofread the DNA sequences they are responsible for synthesizing (14, 15). These proofreading and repair mechanisms must contend with different challenges in different cells, including endogenous mutagens, exogenous mutagens, and the wear and tear that might result from rapid proliferation in certain tissues (16–18). The failure of DNA proofreading and repair can also have different consequences in different tissues—for example, only mutations in germ cells and early embryos can affect the fitness of future generations. For reasons that are not well understood, some tissues are also especially prone to oncogenic transformation, meaning that mutations in these tissues have potentially deadly consequences (19, 20). Conversely, for tissues that are not prone to oncogenic transformation and might tolerate higher mutation rates, it is not clear whether the organism can somehow conserve resources by declining to deploy all the DNA repair mechanisms that are maintained to control mutagenesis in more critical cell types.

In this article, we explore some of the major theoretical frameworks that have been proposed to explain mutation rate differences among cell types, populations, and species. These theoretical frameworks tend to focus on different sources of selective pressure—avoiding germline mutations, avoiding cancer, or avoiding other types of somatic decline with age. They also make different assumptions about the overall effectiveness of selection and the relative role of genetic drift and other constraints in shaping mutation rates. Since theories of germline mutation rate evolution are largely formulated in the language of population genetics, while the somatic mutation rate is also studied from other disciplinary vantage points, we define key population genetics concepts throughout the review. We explore what new kinds of data will likely be needed to distinguish among these frameworks, as well as the human health implications of mutation rate variation among tissues and individuals.

## GERMLINE-DRIVEN THEORIES OF MUTATION RATE EVOLUTION

One theory that posits that DNA repair is primarily tuned to limit heritable mutations is the drift-barrier hypothesis (21–25), an idea rooted in Ohta’s nearly neutral theory (26, 27). The drift-barrier hypothesis assumes that since most new mutations are either deleterious or neutral, it should be selectively beneficial for an organism to minimize the mutation rate and protect the stability of its genome. However, in a species with a small effective population size (*N*_e_) and limited genetic diversity, the ability of natural selection to minimize the mutation rate will hit a barrier, as many mutator alleles (variants that increase the mutation rate) will have fitness effects that are not large enough to overcome the strength of random genetic drift. Lynch (21) previously estimated that in a sexually reproducing species, the selection coefficient of a mutator allele is approximately equal to the total fitness cost of the new mutations that the mutator allele is expected to generate in just one generation. Although the mutator will go on to generate additional deleterious mutations if it remains in the population, most of these will be on other chromosomes and very quickly recombine away, a dynamic that prevents them from contributing to selection against the mutator allele.

The mutation rate may fluctuate over evolutionary time due to the action of drift and selection on such mutator alleles: If it rises too far above the drift barrier, selection against deleterious mutations arising in higher–mutation rate progeny is expected to push the mutation rate back down, whereas if it falls below the barrier, drift will cause it to creep back up. This theory predicts that species with high effective population sizes (such as bacteria, bumblebees, herring, white campion, guinea pigs, and mice) should have lower per-generation germline mutation rates than species with smaller population sizes or highly fragmented populations.

The prediction that the germline mutation rate is inversely correlated with effective population size appears to be empirically supported by germline mutation rates estimated across a wide array of taxa, from prokaryotes to plants (22). A simple example of this is seen when comparing the germline mutation rates of mice and humans: The drift-barrier hypothesis would predict that mice should have a lower per-generation mutation rate than humans, due to their much larger effective population size (28–30), leading to more effective selection against mutator alleles. This prediction appears to hold, with humans incurring approximately two to four times more mutations per generation compared to mice [human mutation rate: 1.2 × 10^−8^ mutations/base pair (bp)/generation, mice: 3.5–5.4 × 10^−9^ mutations/bp/generation (31)]. More generally, a recent study by Bergeron et al. (32) estimated germline mutation rates from family trios across 68 vertebrate species and found a significant negative correlation between per-generation germline mutation rates and effective population size that is not driven by autocorrelation of *N*_e_ across the phylogeny, providing further strong empirical evidence in favor of the drift-barrier hypothesis.

Bergeron et al. also found that low fecundity and long generation times, typical features of low *N*_e_ species, were significantly associated with higher germline mutation rates across taxa. Indeed, many variables beyond *N*_e_ and the efficiency of selection likely contribute to the etiology of mutation rate variation among vertebrates, including the number of germline cell divisions that occur each generation and the tendencies of different developmental stages to be more mutagenic than others (33). In species where each generation is relatively short in terms of both cell divisions and elapsed time, there may be fewer opportunities for mutations to occur even in the absence of especially efficient selection against mutator alleles. At the same time, the first few embryonic cell divisions appear to be consistently more mutagenic than cell divisions occurring later in animal development (34, 35), possibly because these early cell divisions proceed very quickly and the embryo is not yet capable of expressing its full arsenal of DNA repair machinery (36). Thus, a species with a short generation time that experiences more periods of early embryonic cell division per year may accumulate more germline mutations than a species with a longer generation time, a biological quirk that was not modeled in the original formulation of the drift-barrier hypothesis. This feature of early development may be one reason that the mutation rate per year is elevated in mice compared to humans.

An entirely different set of nuances likely influence mutation rate variation among plants, where there is much more prevalent asexual reproduction and no strict sequestration of germline and soma (37). In addition, cancer is a largely unimportant consideration in plant aging, since cell walls tend to confine malignancies in situ and prevent them from metastasizing into anything systemic or life-threatening (38). Because the biology of germline mutagenesis and aging differs so drastically across the kingdoms of multicellular life, we choose to focus this review primarily on animals, where the germline/soma dichotomy is clearest.

## NEUTRALIST VERSUS SELECTIONIST THEORIES OF MUTATION RATE EVOLUTION

The explanatory power of the drift-barrier hypothesis is just one of many testaments to the power of the nearly neutral theory of molecular evolution (27, 39). Although the nearly neutral theory was formulated based on patterns found in relatively small, early datasets, it has been extraordinarily successful at explaining a broad array of differences that exist among large and small populations. One likely reason that this theory has stood the test of time is that it allows for both drift and selection to make important contributions to patterns of genetic diversity, and it simply makes predictions about how effective population size is likely to tune the balance between selection and drift. However, there has always been considerable controversy surrounding the exact balance between drift and selection, including what proportion of human genetic change is neutral as opposed to adaptive (40–46). Such controversy is also evident when we examine competing theories about the evolution of mutation rates, both in the germline and in the soma. While there is no need to choose dichotomously between genetic drift and natural selection as an explanatory theory, either in the context of mutation rate evolution or in the broader setting of evolutionary theory, it can be useful to classify mechanisms of mutation rate evolution into those that predict that the mutation rate will occur close to some selective optimum versus those where genetic drift may be expected to drive mutation rates away from what is optimal.

While the drift-barrier hypothesis is a neutralist theory positing that germline mutation rates are tuned by a combination of genetic drift and stabilizing selection, the theories we classify as selectionist theories focus on enumerating different factors that may influence the optimal value of the mutation rate. One such theory, the cost of fidelity model, posits that the germline mutation rate reflects a balance between the benefits of avoiding deleterious mutations and the energetic cost of avoiding those mutations. In RNA viruses, there exists direct evidence for a trade-off between replication fidelity and replication speed (47–49) (a proxy for virulence), but outside of viruses, it is less clear whether replication fidelity is directly related to proliferation or other traits that might be good proxies for fitness. Although replication fidelity likely always has an energetic cost, the available evidence suggests that this cost explains at most a small amount of the mutation rate variation that exists outside of viruses (25).

While the cost of fidelity model proposes that low mutation rates may incur an indirect energetic cost, the concept of evolvability (50, 51) posits that low mutation rates have a direct second-order fitness cost because they impede populations from adapting to changing environments. Instead of selection favoring the lowest mutation rate possible, in this paradigm, selection favors a higher optimal mutation rate that balances the benefit of avoiding deleterious mutations against the cost of forgoing beneficial mutations. In bacteria, there is evidence that certain mutagenic pathways become upregulated during stressful conditions (52), but it has also been argued that this stress-induced mutagenesis may simply be a side effect of a mechanism that evolved primarily to rescue cells from replication-arresting lesions (53).

Since most mutator and antimutator alleles are likely to have some kind of pleiotropic effects on traits other than germline mutagenesis (including somatic mutagenesis), it is likely always going to be difficult to pinpoint whether changes to the germline mutation rate are driven by direct or indirect selection, even if neutral genetic drift can be rejected as an explanation. This difficulty has been a sticking point during recent debates concerning the interpretation of mutation rate differences between coding and noncoding regions of the genome (54–56). Although we have long known that coding regions tend to have lower mutation rates than noncoding DNA, it is not clear whether the pattern is driven by selection to reduce the mutation rate in conserved regions of the genome or simply induced as a side effect of structural properties such as the packaging of coding and noncoding DNA into different chromatin states (57, 58). In principle, selection for a reduced mutation rate in conserved genomic regions could balance the costs and benefits proposed by the cost of fidelity model. However, such selection does not seem generally consistent with the evolvability model, since mutations in protein-coding genes are the most likely mutations to have beneficial effects, unless the genome is able to evolve higher mutation rates in specific genes that are loci of ongoing positive selection (59).

## CANCER AND LIFE SPAN LIKELY SHAPE THE EVOLUTION OF SOMATIC MUTATION RATES

In animals, only a few cell types (gametes and early embryos) are capable of transmitting their DNA to future generations. Most other cell lineages are destined to live out a finite lifetime within the life span of one individual. Mutations in nongermline somatic cells thus have no effect on population fitness unless they cause the animal to lose fitness by having fewer offspring or providing less parental care. The fitness impact of a high somatic mutation rate is thus highly dependent on an organism’s life span and reproductive strategy, and the fields of geroscience and cancer biology have formalized several theories predicting how somatic mutation rates are likely to diverge in lineages with relevant phenotypic differences (60, 61). If the selective pressures that shape the somatic mutation rate act at odds with the pressures that act on the germline directly, they have the potential to muddy the predictions of models of germline mutation rate evolution.

None of the major germline-focused theories, whether more neutralist or more selectionist, make specific predictions about the impact of selection on somatic mutation rates. However, since many of the same DNA maintenance mechanisms are shared between somatic and germline cells, it has been proposed that strong selection against changes in the germline mutation rate may be accompanied by some amount of concomitant protection against somatic mutations. Evolutionary theories of cancer (61) and aging (60) somewhat complicate this theoretical framework by introducing the idea of selection acting on somatic mutation rates themselves. The somatic DNA damage theory of aging (62) suggests that the lifetime accumulation of damage to somatic cells may contribute to aging, which implies that preventing, repairing, or slowing the accumulation of somatic mutations and DNA damage may be critical to achieving longevity. In particular, long-lived species with large body sizes, which generally have notably low effective population sizes, may have evolved highly robust somatic DNA maintenance mechanisms in order to reduce the incidence of cancer in their multitude of cells and achieve their long life spans (63). The extent of the contribution of nuclear and mitochondrial somatic DNA damage to aging remains uncertain (64), but it is clear that somatic mutations in the nuclear genome do sometimes determine life span by triggering oncogenesis.

Richard Peto, a renowned epidemiologist and statistician, posed a question in the 1970s that is now known as Peto’s paradox: He noted the similarity in cancer rates between mice and humans, species with orders of magnitude differences in their number of cells and life spans (65–70). Peto asked how large, long-lived animals such as humans, elephants, and whales can attain such long life spans without succumbing to cancer, given that cancer occurs at younger ages in some smaller-bodied organisms (71). There are many proposed solutions to Peto’s paradox (69, 72), including expansions of tumor suppression gene families and changes in tissue architecture, immune system processes, and/or rates of apoptosis, but one hypothesis that has captured considerable attention is the possibility that large-bodied and long-lived species tend to evolve more robust DNA surveillance and repair mechanisms. Possible signals of selection for cancer resistance and DNA repair have been documented in the genomes of several long-lived, large-bodied species for which Peto’s paradox is especially relevant, reviewed by Tollis et al. (63). These include an expansion of the *TP53* tumor suppressor gene in elephants (73, 74), signatures of positive selection and differences in gene expression of cancer-related genes in whale lineages (75–78), and possibly greater efficiency of double-strand break repair in bowhead whale fibroblasts (78).

One possible implication of the idea that long-lived species evolve better somatic maintenance mechanisms is that these species may also have evolved lower germline mutation rates (76, 79, 80). This is logical to deduce if long-lived species tend to evolve improved DNA repair mechanisms compared to short-lived species and if these mechanisms tend to be active in the germline as well as the soma. We currently have few measurements of mutation rates in animals that are longer-lived and larger-bodied than humans, but one recent study measured mutation rates of several baleen whale species, some of which can live as long as 200 years. This study estimated an average whale germline mutation rate of 1.11 × 10^−8^ mutations/bp/generation, very similar to rates measured in humans (1.25 × 10^−8^ mutations/bp/generation) (81). These rates are both relatively high among mammals (32, 82), rather than dramatically low in a way that would indicate improved DNA repair affecting both the soma and the germline. Instead, the whales’ mutation rate estimate appears to be consistent with their limited effective population size as predicted by the drift-barrier hypothesis (83)—indicating that whatever may be occurring at the somatic level to prevent cancer in these large long-lived animals is not lowering their germline mutation rates.

The theories that have been proposed to explain the evolution of aging, cancer, and variation in somatic mutation rates also fall along the neutralist–selectionist continuum that we discussed in the context of germline mutagenesis. These theories are reviewed in more depth in References 60 and 61. On the neutralist side, the mutation accumulation hypothesis (84) suggests that genetic variants that cause aging or late-in-life somatic DNA damage might be irrelevant to fitness, falling in a selection shadow after reproduction when purifying selection cannot act to eliminate them, and therefore accumulating in the germline. This selection shadow could be particularly strong if the organism is likely to die at a young age from predation or environmental exposure. An extreme example is the annual turquoise killifish (*Nothobranchius furzeri*), which lives in ponds that disappear every dry season, an environmental niche that imposes a hard upper limit on its life span. Since adaptation to this environment has negated any selection for maintenance of the soma past a single wet season, this killifish species appears to have lost the ability to live more than 4 to 8 months, even in captivity. This short life span has made *N. furzeri* a convenient model organism for the study of aging-related degenerative disorders and cancers (85), which it develops much more quickly than longer-lived organisms do.

The killifish’s rapid aging phenotype may be caused by the relaxation of selection against deleterious variants arising in aging-related genes, including those responsible for genome maintenance (86). Indeed, Cui et al. (86) found that killifish species living in the most extreme, unstable habitats appear to have the least ability to rid their genomes of potentially deleterious transposable element proliferation even compared to sister species that exist at slightly higher population sizes in slightly more stable environments. Nearly 40% of *N. furzeri*’s genes showed a signal of significant relaxed selection, including several genes associated with DNA repair. The genetic drift that occurs during population bottlenecks of killifish in arid regions may also decrease their potential for long life spans (87).

At the opposite extreme from killifish, some life history features of very long-lived species, such as a high degree of parental investment in offspring, can extend selection for longevity significantly past the reproductive life span, as maintaining the parental soma may be critical for the survival of existing offspring. In general, the mutation accumulation hypothesis makes qualitatively similar predictions compared to the drift-barrier hypothesis, as both identify inefficient natural selection as the primary driver of mutation rate differences between species, but they predict strong selection against mutator alleles in very different sets of species, as long-lived species do not often have large effective population sizes.

While neutralist theories of somatic mutation rate evolution posit that mutation rates gradually creep up whenever low mutation rates are insufficiently advantageous, selectionist theories argue that low somatic mutation rates may be actively detrimental to early life success. The theory of antagonistic pleiotropy (88) posits that adaptations promoting early life success are often actively detrimental to longevity, meaning that selection must strike a balance between the two. The disposable soma theory (89) extends the antagonistic pleiotropy framework further by positing that if resources are limited, trade-offs between investing in fecundity and maintenance of the soma may lead selection to favor an early investment in reproduction at the expense of longevity, explaining why short-lived species may have evolved to invest heavily in reproduction rather than longevity. This theory can be extended to predictions about investing in protecting the germline (favoring reproduction) versus maintaining somatic DNA (longevity), which could explain why somatic mutation rates are much higher than germline rates (90).

One intriguing study of human de novo mutation rate variation not only fails to identify a trade-off between fecundity and DNA repair, but actually finds the opposite pattern. In a set of four-generation pedigrees sequenced by the Centre d’Etude du Polymorphisme Humain (CEPH), families with lower-than-average germline mutation rates appear to have longer life spans and greater reproductive success compared to families with higher-than-average germline mutation rates (91). This result suggests that not all alleles promoting longevity and DNA stability are detrimental to early life success. In general, life span variation within species can display different trait correlations and anticorrelations compared to life span variation among species. In another example of this, although *N*_e_ is generally inversely correlated with life span and generation time among species, data from both killifish and *Daphnia* suggest that higher-diversity populations actually have longer life spans than lower-diversity populations of the same species (87, 92). Similarly, although Peto noted that cancer is not especially prevalent in larger-bodied species, within a species body size appears to be positively correlated with cancer risk [including in dogs and humans (93–95)]. It is likely that cancer risk reduction in large-bodied animals evolves relatively slowly, requiring compensatory genetic changes that are separate from the changes that directly cause the body size to increase. We also suspect that the neutralist and selectionist theories of aging are not mutually exclusive, but that shorter life spans are created by a combination of genetic drift and selective trade-off with reproduction. Within populations, genetic drift may play a bigger role than selection, leading to the trends observed within killifish and *Daphnia*, whereas selection likely drives a proportionally larger proportion of genetic change over longer evolutionary timescales.

## WHICH PREDICTIONS OF CLASSICAL MODELS ARE SUPPORTED BY SOMATIC MUTATION DATA?

Distinguishing among neutralist, selectionist, germline-driven, and somatic-driven models will ultimately require data on somatic mutation accumulation across the life spans of short-lived and long-lived animals. As shown in Supplemental Table 1, somatic mutation rate measurements are currently taxonomically sparse; cutting-edge techniques such as single-cell genomic analysis and microdissection of clonal tissues are new enough that they have not yet been applied to many species. Cagan et al. (96) recently found that the somatic mutation rate in colon crypts appears to be negatively correlated with life span—long-lived species such as humans appear to have lower somatic mutation rates than short-lived species such as mice, to the extent that humans and mice accumulate similar burdens of mutations in their colon epithelia by the ends of their respective life spans. A parallel study (97) of comparative mutagenesis in hematopoietic stem cells only partially echoed these conclusions—although this study also found a higher mutation rate per year in mice compared to humans, it still found that human hematopoietic stem cells accumulated many more mutations by the natural end of life. This hematopoietic stem cell result echoes the dynamics of the germline, where the mouse mutation rate exceeds the human rate when measured per year but is lower than the human rate when measured per generation.

Mice, humans, and whales are such distantly related species that it is difficult to give a conclusive reason why mutation rates may have evolved differently along their respective lineages (98). It is perhaps more informative to look at mutation rate variation among more closely related species to determine whether it tends to be correlated with recent changes in life span, population size, or other relevant traits (98). For example, Tian et al. (99) found that longer-lived rodents (such as beavers) have more efficient orthologs of SIRT6, a tumor suppressor responsible for sensing double-strand DNA breaks, compared to shorter-lived mice. Similarly, Zhang et al. (100) used single-cell whole genome sequencing across two strains of mouse, guinea pig, blind mole rat, naked mole rat, and human lung fibroblasts to characterize somatic mutation rates with and without exposure to a mutagen. They found that the shortest-lived species, the mouse, had a markedly higher (approximately twofold) spontaneous somatic mutation rate per cell division in culture, which they hypothesized could be due to differences in the mouse polymerase η (*Polh*) gene. However, they found no significant differences among the spontaneous mutation rates in the four other longer-lived species.

While Zhang et al. found spontaneous mutation rates in vitro to be largely consistent across species, aside from mice, they reported a more robust negative correlation between the somatic mutation rate and species life span in cells treated with the mutagen bleomycin. This suggests that longer-lived species may have more robust DNA repair mechanisms, but they do not always have lower spontaneous mutation rates in the absence of environmental perturbation. These studies provide tantalizing molecular evidence for the idea that somatic DNA replication and repair are more effective in longer-lived species, but they ultimately provide very little power to distinguish among neutralist and selectionist mechanisms for the evolution of such differences.

One pattern that is emerging from modern mutation data is that life span is a better predictor than body size of both germline and somatic mutation rates across species. Life span has a greater impact on the number of total stem cell divisions than body size, which may help to explain why longevity is the more impactful of the two life history traits for the evolution of somatic mutation rates and cancer risk (98). Tollis et al. (101) characterized cancer gene expansions across mammals and found a significant association between longevity quotient (life span relative to body size) and cancer gene expansions, but no such association with body size. Cagan et al. (96) found life span to be a better predictor of the somatic mutation rate than body size, and discussed how the lack of a significant anticorrelation between somatic mutation rates and body size may indicate that large-bodied species are not reducing their cancer risk by lowering their somatic mutation rates, but are instead employing some other evolutionary strategy.

It is notable that the species with the highest somatic mutation rates in the Cagan et al. study (rats and mice) are species with short life spans and rapid reproductive strategies. Their relatively high somatic mutation rates thus line up with the predictions of the disposable soma theory of aging, but this finding contradicts the expectations of the drift-barrier hypothesis given mice’s and rats’ large effective population sizes in the wild. This contradiction is straightforward to resolve if we suppose that there is not much pleiotropy between germline and somatic mutation rates, which is the same resolution we proposed to explain the relatively high germline mutation rate of whales.

### Disentangling the Effects of Population Size from Generation Time and Phylogenetic Inertia

In a larger study that measured germline mutation rates across diverse mammals, Bergeron et al. (32) did not find a significant association of mutation rate with either body size or longevity. This lack of association might follow from the fact that the germline is sequestered early in development and its cell count does not tightly correlate with the total cell count of the mature animal. The lack of correlation between longevity and germline mutation rate is perhaps more surprising: If long-lived organisms do evolve more effective somatic DNA repair as suggested by Peto’s paradox and several empirical studies, it is not clear why this more effective repair machinery fails to lower the mutation rate in the germline.

**Figure 1:**
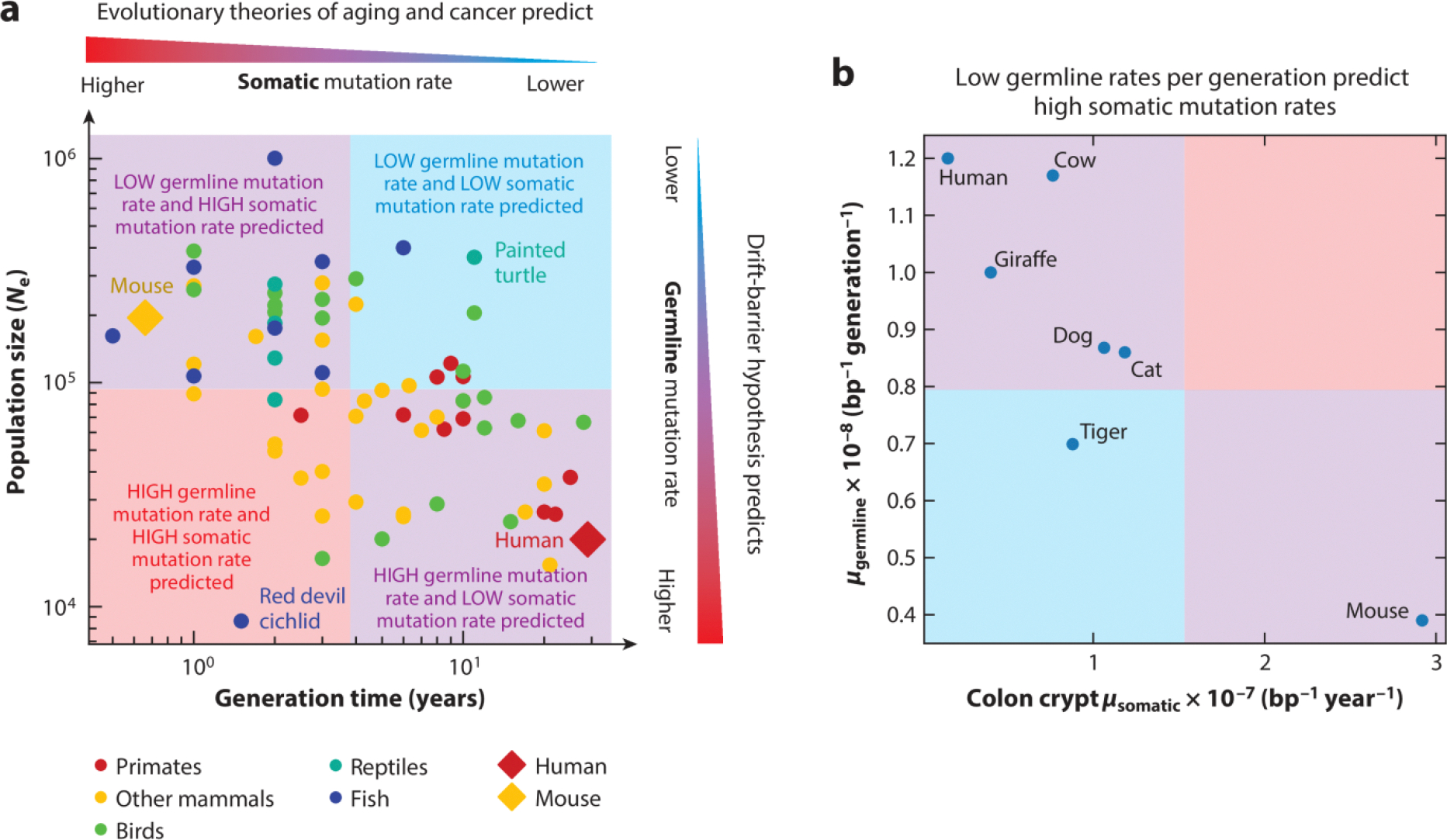
Theories of germline and somatic mutation rate evolution often predict conflicting trends. (*a*) Vertebrate species display the inverse correlation between generation time and *N*_e_ that was previously noted by Chao & Carr (103) across a broader set of species. This implies that most species fall into life history parameter regimes where theories of germline and somatic mutation rate evolution make conflicting predictions (*purple boxes*). Relatively few species fit into parameter regimes where existing theories predict that somatic and germline mutation rates should be consistently high or low (*red* or *blue box*, respectively). Data from Wang & Obbard (82); see also Supplemental Table 2. (*b*) In a set of seven vertebrate species for which we have comparable germline and somatic (colon crypt) mutation data (96), germline mutation rates (*μ*_germline_) per generation are inversely correlated with somatic mutation rates (*μ*_somatic_) per year. The colored boxes indicate regions of the graph where somatic and germline rates are both higher (*red*), both lower (*blue*), or inconsistent with each other (one high, one low; *purple boxes*). Data from Wang & Obbard (82) and Cagan et al. (96). Abbreviation: bp, base pair.

The well-known inverse correlation (102, 103) between the two life history variables generation time and effective population size can be seen in the vertebrate species included in a study by Wang & Obbard (82) (Figure 1a). This relationship may exist because long-lived, late-reproducing species require more resources per capita and may not have enough environmental resources to achieve large population sizes. This inverse correlation implies that most species occupy life history regimes where theories of somatic and germline evolution make conflicting predictions—mice and rats are not at all exceptional in having short generation times combined with large effective population sizes.

The phenotypic similarity between species caused by shared histories must always be accounted for when estimating the correlations between traits such as mutation rate, effective population size, and generation time. Methods such as phylogenetic independent contrasts, phylogenetic generalized least squares (PGLS), and phylogenetic generalized linear mixed models (PGLMMs) (104–108) allow for phylogenetic corrections to regressions, enabling principled joint modeling of trait covariation across a phylogenetic tree. Although *N*_e_ is a trait with significant phylogenetic signal, with a tendency to be higher in microorganisms and lower in long-lived multicellular organisms, a recent PGLMM analysis has so far found that *N*_e_ is able to explain additional mutation rate variation on top of what is explained by phylogenetic inertia and generation time effects (82).

### Comparison of Somatic and Germline Mutation Rates Within and Between Species

When comparing germline and somatic mutation rates among the seven species for which mutation rates have been measured in both the germline and the colon epithelium (Figure 1b), we see an inverse correlation between the germline mutation rate per generation and the somatic mutation rate per year, echoing what we saw when comparing mice and humans (PGLS *p* = 0.013), with the caveat that the strength of the phylogenetic signal in this dataset cannot be reliably estimated from just seven samples (see the section titled Methods for details). Among these species, somatic and germline mutation rate variation conforms to the marginal predictions of the disposable soma and drift-barrier hypotheses, but together they do not paint a coherent picture of certain species having systematically more effective DNA repair than others.

Although all cells have access to the same DNA repair machinery, different human cell types have mutation rates that differ by at least an order of magnitude (5) (Supplemental Table 1). One recent study calculated that in both mice and humans, skin fibroblasts accumulate nearly 100 times as many mutations per cell division as germline cells (109). Mutation rate differences among cell types are often accompanied by differences in mutational spectra (5), indicating that the mutations are created by different combinations of DNA damage and repair processes. Many of these mutation spectrum differences were previously discovered in the context of cancer, but their presence in normal tissues implies that they are actually physiological rather than pathological.

If selection favors low mutation rates in certain cells such as germ cells, what might prevent other cells from utilizing shared DNA repair machinery to achieve similarly low mutation rates? One possibility is that germline and somatic cells rely preferentially on different DNA repair pathways; for example, germ cells likely do not rely heavily on the UV light repair mechanisms that are essential for avoiding cancer-causing mutations in skin cells. Cell types also vary in their exposure to endogenous mutagenic processes, including processes such as methyl-CpG deamination that appear sensitive to the rate of cell division. It is also unclear how costly DNA repair might be in terms of energy and resources—if costs are high, this might favor repression of highly accurate DNA repair machinery in cells where this machinery has little selective benefit.

Even if deploying DNA repair is not especially costly, genetic variation in regulatory regions might create differences in the expression of DNA repair genes, which could in turn cause mutation rates to differ between tissues. If such variation happens to repress DNA repair gene expression in somatic tissues where higher mutation rates are tolerated, genetic drift might cause these variants to reach high frequencies even though they are slightly deleterious. There is some empirical evidence for the existence of such variation: A mutator allele recently identified in laboratory mice appears to act by reducing the expression of the DNA repair gene *Mutyh* (110–112). It is important to note that nearly all mouse and rat studies, including Cagan et al.’s (96) study, have utilized inbred laboratory strains that are maintained at extremely low effective population sizes, which could facilitate the drift of mutator variants to high frequency (110–112). This effect might also explain Zhang et al.’s (100) finding that laboratory mice are a fast-mutating outlier among rodents. In Supplemental Table 1, we have attempted to comprehensively record existing mutation rates measured to date from different germline and somatic tissues, excluding certain cell-line-based measurements that are not suitable for comparison to other measurements because they have not been scaled as mutations per cell division or another comparable time unit.

The mutation rate differences observed among human and mouse cell types appear to validate several predictions of classical theories of aging. As long predicted, higher mutation rates are observed in highly replicative epithelial tissues that are known to be prone to cancer (though tissues such as the brain with lower mutation loads are not immune to oncogenic mutations) (5). One tissue with an extremely high mutation rate is the placenta, which lives for the duration of gestation before being expelled from the body (113). The placenta is also an organ that proliferates very quickly and is exposed to waste and oxidative stress through its role in gas and nutrient exchange, but its rapid mutation accumulation suggests that the efficiency of DNA repair can vary among tissues as a function of their longevity, providing support for the disposable soma theory at a per-tissue level.

Although germline and somatic mutational processes are clearly biologically distinct, these differences are also amplified by a discrepancy in how these rates tend to be reported. Germline mutation rates are usually scaled per generation because they are measured by comparing parents and offspring. In addition, selection fundamentally operates on the mutation load per generation, since this is the load that determines offspring fitness. In contrast, somatic mutation rates are scaled in more variable ways: sometimes per cell division or per year and occasionally as an end-of-life burden.

To illustrate the impact of mutation rate scaling on our interpretation of the data, we revisit the inverse correlation between germline and somatic mutation rates plotted in Figure 1b. We can make these germline mutation rates more comparable to somatic mutation rates by transforming them into rates measured per cell division or per year, and the most appropriate choice of rescaling depends on the etiology of germline mutagenesis. Per–cell division scaling is appropriate assuming that most mutations arise as copying errors during mitosis, whereas per-year scaling is more appropriate if most mutations are caused by DNA damage and arise independently of cell division.

Although germline mutations were once thought to stem from DNA replication errors occurring in spermatocytes and the early embryo, several lines of evidence now suggest that germline mutagenesis does not actually track cell divisions, but accumulates due to replication-independent DNA damage in quiescent female gametes as well as proliferating male gametes (114, 115). One recent study estimated that CpG transitions do track cell divisions in the germline, but it also estimated that about 90% of germline mutations accumulate at a rate proportional to time rather than a rate proportional to cell division (33). In species that lack CpG methylation in the germline, it is likely that not even mutations at CpG sites occur at a rate proportional to cell divisions, so for simplicity we will rescale each species’ overall mutation rate from per generation to per year, neglecting the small proportion whose etiology is different.

**Figure 2:**
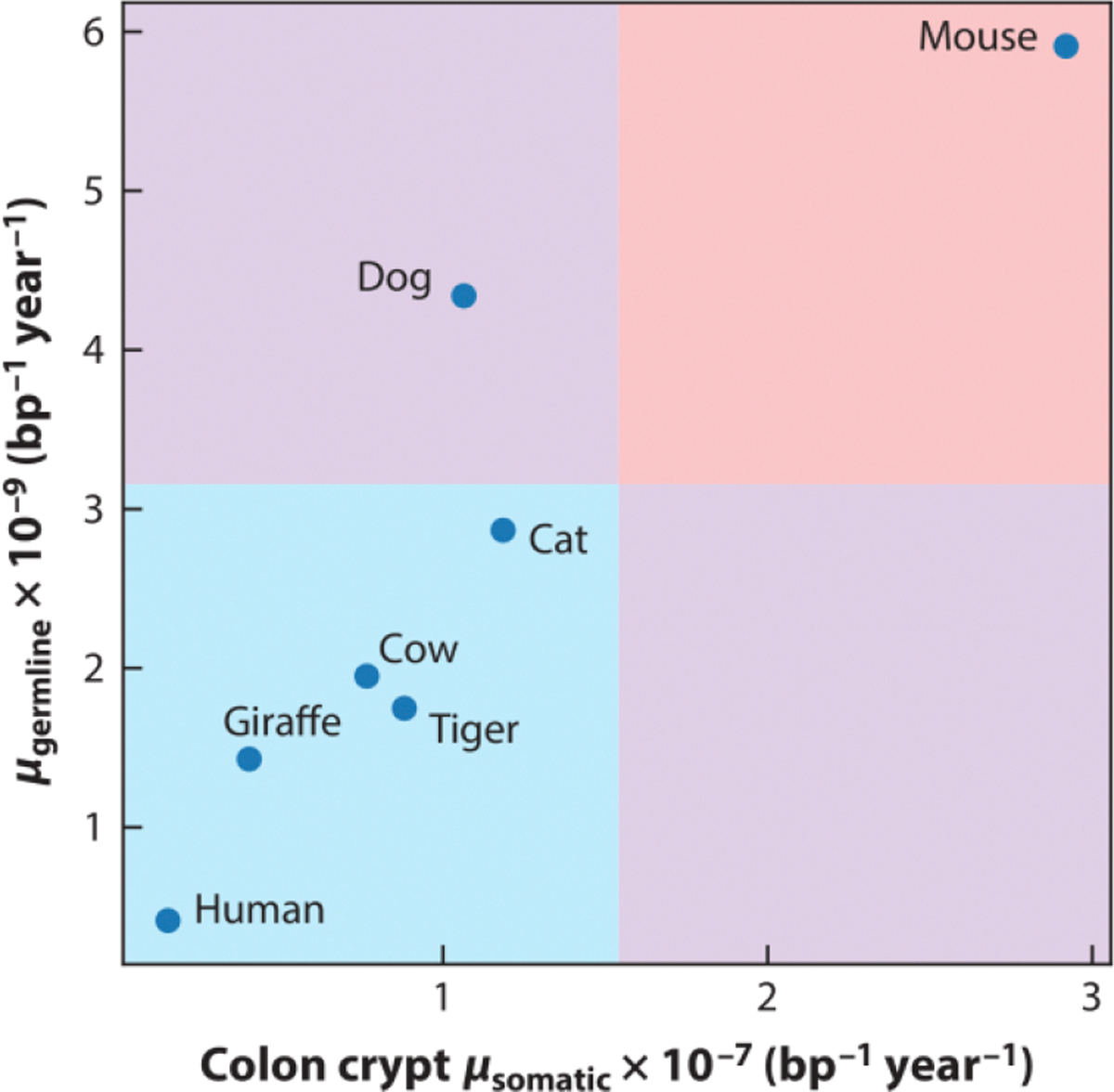
Fast-reproducing species have consistently high mutation rates across cell types. Germline mutation rates (*μ*_germline_) and colon crypt (somatic) mutation rates (*μ*_somatic_) appear positively correlated when both are measured per year. The colored boxes indicate regions of the graph where somatic and germline rates are both higher (*red*), both lower (*blue*), or inconsistent with each other (one high, one low; *purple boxes*). Data from Wang & Obbard (82) and Cagan et al. (96). Abbreviation: bp, base pair.

If we rescale the germline mutation rates from per generation to per year, the inverse correlation between the mutation rate and *N*_e_ actually reverses and becomes a positive correlation (PGLS *p* = 0.007) (Figure 2). The same trend has been seen in multiple studies of different human and mouse cell types: Mutation rates per cell division are consistently higher in mice than in humans (31, 97, 109). Mathematically, this reversal can be explained by the negative correlation between *N*_e_ and generation time: When we divide the mutation rate per year by the length of a generation, these generation lengths are systematically larger in high-*N*_e_ species. Although we can argue that mutations per generation is the correct unit for measuring the impact of the mutation rate on fitness, we could also argue that mutations per year is a better unit for comparing the efficacy of DNA repair across species.

One question for which we have almost no empirical data is whether the additional mutations caused by mutator alleles tend to accumulate at constant rates measured per year, per cell division, per generation, or per some other time unit. Even for the small number of mutator alleles that have been identified using mutation data in human families affected by cancer syndromes or large colonies of laboratory organisms, we lack the appropriate data to measure whether these mutations are accumulating during cell divisions or during specific periods of passive cellular aging. We might extrapolate from the per-year accumulation of most germline mutations that germline mutator effects are most likely to scale per year as well—if we do so, this allows us to extrapolate that mutators have stronger fitness effects in species with long generation times, a modification to the drift-barrier hypothesis that brings its predictions more in line with the predictions of the disposable soma theory (116).

The extent of pleiotropy between germline and somatic mutation rates is a topic that bears revisiting as more detailed data become available. Above, we proposed that conflicting predictions of germline and somatic theories might be reconciled by a lack of pleiotropy between germline and somatic mutation rates, but at face value, the tight correlation between germline and somatic rates measured per year does appear to imply that species with more effective germline DNA repair also have more effective somatic DNA repair. We infer two possibilities that are difficult to distinguish given the current data: Either DNA repair efficacy exhibits little pleiotropy between the tissues and the correlation in Figure 2 is driven by the correlation of *N*_e_ and generation time, or low-*N*_e_ species have consistently more effective DNA repair than high-*N*_e_ species, perhaps due to biological factors that were not incorporated into drift-barrier calculations that made the opposite prediction. More joint estimates of germline and somatic mutation rates from the same species, in interpretable, comparable units, will be essential to deduce the true nature of pleiotropy between germline and somatic mutation rates as well as their relationship to effective population size and life span. We argue that the data most sorely needed are data from species with very different life histories, spanning a range of values of life span and *N*_e_, and emphasizing outliers that fill out the full range of life span and population size covariation.

## HOW HERITABLE IS MUTATION RATE VARIATION IN NATURAL POPULATIONS?

If selection is acting to tune mutation rates in response to factors like body size or life span, the speed of this adaptation will depend on the mutational target size: How often do new mutations affect the mutation rate and how large do these impacts tend to be? Even if a lower mutation rate is favored by evolutionary changes such as the expansion of body size or the lengthening of life span, lower mutation rates cannot actually evolve in the absence of genetic variation that has this phenotypic effect.

Some traits, like height, are both highly heritable and highly variable within and between populations. This standing genetic variation facilitates rapid evolution in response to changing selective pressures—as soon as taller or shorter height becomes favored by a shift in the environment, there is a ready pool of height-modifying alleles for this new selective pressure to act upon. In contrast, if heights over 6 feet tall were initially subject to strong negative selective pressure that eliminated all variants associated with extreme height, but became selectively favored after an environmental shift, such adaptation could not proceed until height-increasing alleles had time to arise de novo and establish within the population. Since very little is currently known about the heritability of mutation rates within the human species or other populations, it is difficult to assess how quickly the mutation rate will be able to respond to changes in life span or other traits that alter the selective cost of a high mutation rate.

Despite our lack of knowledge about which mutations are able to change the mutation rate, the empirical inverse correlation between mutation rate and effective population size provides some evidence that most populations likely harbor some nearly neutral mutator variation, at least if this correlation is driven by the mechanism put forward by the drift-barrier hypothesis. The reason that the drift-barrier hypothesis predicts higher mutation rates in low-*N*_e_ populations is that a higher proportion of mutator alleles is likely to be nearly neutral in these populations. In a population where more mutator alleles are nearly neutral in their fitness effects, a higher proportion of mutator alleles should rise to high frequency or even fix than would be the case in a population that had been purged of such variation.

Of course, a small population might still have a low mutation rate if the mutator allele target size were very low and no mutation rate–increasing variation happened to rise to appreciable frequencies. In practice, however, empirical mutation rate estimates tend to stay within five- to tenfold of the prediction of a regression against effective population size (117). This correlation suggests that all populations (except perhaps those of extremely high *N*_e_) contain mutator variation that causes their mutation rates to be higher than the mutation rate expected of a larger-*N*_e_ population. Moreover, regressions of the mutation rate versus *N*_e_ do not show evidence of a critical value of *N*_e_ above which the correlation with the mutation rate breaks down because the mutation rate has reached some theoretical minimum. This pattern suggests that most populations contain standing mutator variation that may be selected upon when body size or life span changes, allowing the mutation rate to change as predicted by selectionist theories.

One recent study that sequenced 1,465 human trios was unable to exclude the possibility that germline mutation rate variation was entirely determined by environmental variance and random sampling noise (118)—in the absence of more powerful studies, it is hard to estimate how quickly the mutation rate may adapt in response to other phenotypic change. Two factors that limit the power of even very large de novo mutation studies are the noisy nature of mutation rate measurements and the strong confounding effect of reproductive age—as previously discussed, this can create nonheritable variation in the germline mutation rate, even among families within the same species (119), and likely across species as well (120). A third factor is the existence of at least weak selection against high mutation rates, which likely ensures that most mutation rate–increasing variation is rare, recessive, or of small effect size.

Although only a handful of mutator alleles have been identified to date and we have no precise estimates of the human mutation rate’s heritability, there are several indirect indications that this heritability is likely to be nonzero. In a much simpler system, the BXD family of recombinant inbred mice, an artificial breeding structure has facilitated the direct measurement of nonzero mutation spectrum heritability (112). In a more natural but still restricted setting, the human CEPH pedigree collection, significant mutation rate variation independent of parental age has been measured among families (121). Outside of these special settings, perhaps the strongest indication that germline mutation rates are heritable is the existence of mutation spectrum variability among diverse populations and species. As human populations have diverged over just a few tens of generations, each continental group has developed its own characteristic mutational spectrum as measured by the distribution of sequence contexts where its polymorphisms tend to occur (122, 123). Although some of this variation might be due to nongenetic causes such as generation time differences among populations (124, 125), several lines of evidence indicate that generation time alone is likely not adequate to explain the observed variability, making genetic variation a more likely explanation (126, 127). Similar patterns have since been observed over a variety of longer timescales in different groups, including the great apes (128), a diverse collection of mammals (129), an ancient phylogeny of *Saccharomyces cerevisiae* (130), and the recently emerged variant clades of the virus SARS-CoV-2 (131). Mutation spectrum divergence generally appears to scale with phylogenetic divergence, a pattern expected of a polygenic trait (129).

Given the strength of the evidence for heritability of germline mutational processes, our failure to identify many underlying genes can be seen as a special case of the missing heritability problem that is known to affect many genome-wide association studies of modest sample size, particularly when traits are known to be under stabilizing selection. For certain well-studied traits, the missing heritability problem has largely been solved as decreases in genotyping and sequencing cost have enabled larger and better designed studies (132). New methodology is also being designed to mitigate some of the unique sources of noise that have hurt the power of early mutation rate association studies (112).

## CONCLUSIONS AND OUTLOOK

Unifying evolutionary theories of germline and somatic mutagenesis based on population genetics, cancer, and aging biology is an important theoretical concern. It is likely that many intrinsic and extrinsic factors, including population size, generation time, life span, body size, extrinsic mortality, investment in offspring, stochasticity, and environmental mutagen exposure, will be required to precisely model mutation rate variation among species, within species, and within the cells of an individual. It is becoming increasingly clear that a wide array of life history and environmental forces may shape the evolution of life span—for instance, high extrinsic mortality, classically thought to drive the evolution of shorter life spans, can actually lead to the evolution of longer life spans under some conditions (60). It appears similarly likely that no single set of rules can perfectly predict mutation rate variation across taxa, given the complex interplay between life history and environmental forces.

Despite the simplicity of classical mutation rate evolution theories, many of which predate the availability of relevant data, models such as the drift-barrier hypothesis and the disposable soma theory have predicted many of the high-level trends we are discovering by directly measuring germline and somatic mutagenesis. The drift-barrier hypothesis also provides an explanation for the heritable variation exhibited by mutation rates and why it has been so difficult to identify the loci that drive this heritability. On the somatic side, the disposable soma theory appears to explain why somatic mutation rates correlate so strongly with the age of the organism and (in the case of the placenta) with the longevity of the particular tissue. One pattern that is emerging is that somatic-focused theories do a good job of predicting how somatic mutation rates vary as a function of life span, tissue longevity, and tissue cell division rate but do a poorer job of predicting germline mutation rate trends. For example, it does not appear that whales’ extreme longevity has caused them to evolve a lower germline mutation rate than has been observed in other vertebrates (81, 83). Conversely, the drift-barrier hypothesis predicts a negative relationship between germline mutation rate and effective population size, and while this trend can be seen in empirical mutation rates scaled per generation, the prediction of more efficient DNA repair in high-effective-population-size species does not appear to translate to lower somatic mutation rates in high-diversity species, at least when mutation rates are measured per year or per cell division (96).

This failure of somatic theories to translate to the germline and vice versa may mean that pleiotropy between DNA proofreading and repair in different tissues is not especially high. Although all cells in the body utilize the same tool kit of DNA proofreading and repair mechanisms, it appears that different cell types differ widely in their utilization of these tools, unless the observed differences in mutation load can be attributed primarily to mutagen exposure. Another possibility is that species with long generation times have consistently better DNA repair, as suggested by their consistently low mutation rates per cell division, but this would require a revision of the drift-barrier interpretation of the correlation between the germline mutation rate and *N*_e_.

We close by noting that the conclusions of this article are the result of extrapolations from a few tissue samples across even fewer species. Given the current rapid pace of technology development for measuring mutation rates, we expect much more data to become available in the coming years, which may either refute or confirm the various trends we have laid out here. We expect that such data generation will require a combination of new sequencing methodologies and new bioinformatic analysis techniques: Although researchers have now spent a decade identifying mutations using short-read next-generation sequencing data, germline mutation calling is still more an art than a science, requiring time-intensive, error-prone manual correction to separate true signal from sequencing error (133). It is not uncommon for different methodologies to estimate very different mutation counts from the same short-read data (134), and there are unexplained discrepancies between the spectra of mutations measured de novo and the mutations segregating in populations at very low frequencies (125, 135). Long-read sequencing has enabled higher-accuracy mutation calling, even in repetitive regions of the genome that were inaccessible for mutation calling using short-read methods, but the cost of long reads must fall to make this approach scalable to a diversity of organisms (136).

Somatic mutations are especially challenging to distinguish from sequencing errors—accurate measurements of such mutations currently require technologies such as very high-depth sequencing of both strands of each DNA duplex (137) or labor-intensive amplification of single-cell lineages in cell culture (138). Measuring more somatic mutations at scale will require either a reduction in the cost of such technologies or the development of bioinformatic techniques that are able to improve the accuracy of somatic mutation calling in more affordable data types such as bulk RNA-seq (139, 140) or low-coverage single-cell sequencing (141, 142). Computational clonal deconvolution methods designed for inferring the mutational histories of tumors also show promise for use in normal tissues (143–145).

As a complement to better mutation detection methods, numerical and computational models of how life history traits such as population size and life span may influence mutation load may help us generate additional testable hypotheses for analyzing existing and future mutation data (60, 146, 147). Population genetics simulations are now able to incorporate life history traits, changes in the mutation rate, and shifts in selective pressures (148), enabling increasingly complex evolutionary models to be simulated and tested.

We expect that new germline and somatic mutation data will not only help us measure the effects of life history variables, but will also be instrumental for detecting more of the mutator alleles that putatively cause mutation rates to vary within and between species. Although a small number of mutator alleles have already been discovered (110, 112, 130, 149–153), these variants cannot explain a measurable amount of the mutation rate variation recorded in Figures 1 and 2. If we are able to determine the main genetic factors that cause mutation rates to vary within and among species, it will be possible to directly investigate how these genes vary in their activity among tissues and stages of development. This will help us determine how coupled germline and somatic mutational processes are, the relative importance of DNA damage versus replication errors, and the interaction of genetic mutators with selection and life history traits.

By highlighting regimes of life span and population size where classical theories run into the most conflict, we hope to encourage the community to prioritize these parameter regimes for more intensive study, which will likely require a combination of new data generation and new data analysis techniques. As long as so much of our understanding of the evolution of cellular housekeeping processes is based on data from humans and laboratory mice, we will have limited ability to predict how the integrity of our genomes will respond to the myriad niches that organisms adapt to outside of the histories of these species.

## METHODS

Somatic mutation rates from Cagan et al. (96) (provided in the unit of mutations/genome/year, corrected for the analyzable genome size) were rescaled to the unit of mutations/bp/year by dividing rate estimates by the total genome size. Germline mutation rates from Wang & Obbard (82) were rescaled to the unit of mutations/bp/year by dividing rate estimates by generation times (years). *N*_e_ was estimated using the formula *N*_e_ = *π*/4*μ*, where *π* is the neutral genetic diversity and *μ* is the germline mutation rate. Total genome size, generation time, and germline mutation rate estimates were all obtained from Wang & Obbard (82).

We performed PGLS regression on the germline versus somatic mutation rate dataset (Figures 1b and 2) using the R library caper (154). The phylogenetic tree from Wang & Obbard (82) was used as an input to estimate the covariance matrix. Pagel’s λ (155) was used to quantify the amount of phylogenetic signal in the dataset and was estimated using the maximum likelihood (ML) implementation from caper. In short, λ is an internal branch length scaling parameter that ranges from 0 to 1, and a higher λ value suggests stronger phylogenetic signal in the dataset. Although λ_ML_ = 0, we failed to reject the two null hypotheses λ = 0 and λ = 1, indicating high uncertainty about the amount of phylogenetic signal in this small dataset.

## Supplementary Material

Supplemental Table 1

Supplemental Table 2

## Data Availability

The datasets used in our analyses are provided in Supplemental Tables 1 and 2. The germline mutation rate estimates are adapted from Reference 82 by averaging mutation rates for the same species and filtering for species with estimates for both genetic diversity and the germline mutation rate.

## SUMMARY POINTS

1. Mutation rates vary among populations, species, and somatic cell types because of different selective pressures and different amounts of genetic drift.
2. Compared to germline mutation rates, somatic mutation rates have been measured in many fewer species due to a combination of higher sequencing costs and greater data analysis challenges.
3. Germline mutation rates are predicted to be inversely correlated with genomic diversity due to the nearly neutral theory of population genetics, and they might also be influenced by other selective pressures such as the need to maintain evolvability.
4. Somatic mutation rates are predicted to be inversely correlated with life span since the integrity of the soma only needs to be preserved over the organism’s natural life span.
5. Since genetic diversity tends to be highest in species with short life spans and short generation times, long-lived species are puzzlingly predicted to have low replication fidelity in the germline yet high replication fidelity in somatic tissues.
6. It is very difficult to identify genes that underlie mutation-related phenotypes, but identification of more such genes will likely help us refine theories about why mutation rates vary among species and tissues.
7. To resolve the conflict between the predictions of theories of somatic and germline mutation rate evolution, we need more mutation rate data from the germline and especially the soma, which will require improvements in both sequencing technologies and bioinformatic analysis techniques.

## Abbreviations

Mutation rate (*μ*): the rate at which mutations arise in the genome, measured in the germline or in a particular somatic cell type
Germline: the lineage of cells that are transmitted from generation to generation, including gametes and early embryonic tissues that precede the sequestration of the germline
Genetic drift: evolutionary change caused by random chance rather than selection, e.g., when a natural disaster kills a genetically fit individual
Nearly neutral theory: an extension of the neutral theory that posits that most mutations have slightly deleterious impacts, weaker than genetic drift
Effective population size (*N*_e_): an estimate of population size based on genetic diversity; it is equal to the census size in certain unstructured populations
Mutator allele: a mutation that increases the mutation rate in the germline and/or soma, e.g., by reducing the efficacy of DNA repair
Selection coefficient (*s*): a coefficient (|*s*| < 1) that measures the impact of a mutation on fitness; a mutation with *s* = −0.01 decreases fitness by 1%
Generation time: the age at which an individual produces offspring—average generation times can vary widely within and between taxa
Soma: the entirety of an organism’s body—most somatic cell lineages cannot transmit genetic material to offspring but die when the organism dies
Neutralist theory: an evolutionary theory that posits that neutral genetic drift causes the majority of genetic change and natural selection has a smaller impact
Stabilizing selection: selection against phenotypic outliers in favor of a stable trait range
Positive selection: selection in favor of new beneficial mutations
Purifying selection: selection against new deleterious mutations
Selectionist theory: an evolutionary theory in which genetic change is primarily driven by natural selection rather than drift
Heritability: the proportion of phenotypic variation caused by genetics (as opposed to environment or randomness)
